# Optimized procedures for testing plasma metanephrines in patients on hemodialysis

**DOI:** 10.1038/s41598-021-94104-9

**Published:** 2021-07-19

**Authors:** Christina Pamporaki, Aleksander Prejbisz, Robert Małecki, Frank Pistrosch, Mirko Peitzsch, Steffen Bishoff, Petra Mueller, Iris Meyer, Doreen Reimann, Katarzyna Hanus, Andrzej Januszewicz, Stefan R. Bornstein, Simon Parmentier, Carola Kunath, Jacques W. M. Lenders, Graeme Eisenhofer, Jens Passauer

**Affiliations:** 1 Department of Medicine ΙΙI, University Hospital Carl Gustav Carus at the TU Dresden, Fetscherstraße 74, 01307 Dresden, Germany; 2 National Institute of Cardiology, Warsaw, Poland; 3 Department of Nephrology, Centre of Uronephrology, Międzylesie Clinical Hospital, Warsaw, Poland; 4 Dialysis Center Hoyerswerda, Dresden, Germany; 5 Institute of Clinical Chemistry and Laboratory Medicine, University Hospital Carl Gustav Carus at the TU Dresden, Dresden, Germany; 6 Nephrologische Gemeinschatpraxis, Dresden, Germany; 7 Dialysis Center Heidenau, Heidenau, Germany; 8 Kidney/Hypertension/Rheumatology Center, Dresden, Germany; 9 Department of Internal Medicine, Radboud University Medical Center, Nijmegen, The Netherlands

**Keywords:** Biomarkers, Endocrinology, Medical research, Nephrology

## Abstract

Diagnosis of pheochromocytomas and paragangliomas in patients receiving hemodialysis is troublesome. The aim of the study was to establish optimal conditions for blood sampling for mass spectrometric measurements of normetanephrine, metanephrine and 3-methoxytyramine in patients on hemodialysis and specific reference intervals for plasma metanephrines under the most optimal sampling conditions. Blood was sampled before and near the end of dialysis, including different sampling sites in 170 patients on hemodialysis. Plasma normetanephrine concentrations were lower (*P* < 0.0001) and metanephrine concentrations higher (*P* < 0.0001) in shunt than in venous blood, with no differences for 3-methoxytyramine. Normetanephrine, metanephrine and 3-methoxytyramine concentrations in shunt and venous blood were lower (*P* < 0.0001) near the end than before hemodialysis. Upper cut-offs for normetanephrine were 34% lower when the blood was drawn from the shunt and near the end of hemodialysis compared to blood drawn before hemodialysis. This study establishes optimal sampling conditions using blood from the dialysis shunt near the end of hemodialysis with optimal reference intervals for plasma metanephrines for the diagnosis of pheochromocytomas/paragangliomas among patients on hemodialysis.

## Introduction

Pheochromocytomas and paragangliomas (PPGLs) are neuroendocrine tumors derived from the chromaffin cells of the adrenal medulla or extra-adrenal chromaffin tissue^1^. Although rare, these tumors constitute an important endocrine cause of hypertension^2^. Current clinical practice guidelines stipulate that biochemical screening for PPGLs should include measurements of either plasma free or urinary fractionated metanephrines^3^. Additional measurements of 3-methoxytyramine are useful for identifying occasional tumors that predominantly produce dopamine^4^.

In patients with advanced renal insufficiency, the diagnostic work up of PPGLs is troublesome. Similar to patients with PPGLs, many patients with end-stage renal disease (ESRD) on hemodialysis (HD) suffer from hypertension with wide swings in blood pressure. Biochemical confirmation or exclusion of PPGLs in such patients is confounded by the effects of impaired renal function on the elimination of catecholamines and their metabolites in urine^5,6^. However, as the circulatory clearance of plasma free metanephrines is relatively independent of renal function^7^, measurements of these compounds in plasma might be preferred in patients with ESRD. Yet, increases of plasma free metanephrines have been reported in up to 25–28% of patients on HD^8–10^, possibly reflecting CKD-associated activation of the sympathetic nervous system^11,12^.

We have recently published cut-offs for normetanephrine (NMN), metanephrine (MN) and 3-methoxytyramine (3-MTY) plasma concentrations with the use of liquid chromatography-tandem mass spectrometry (LC–MS/MS) in patients with CKD stage III, IV from morning peripheral venous blood samples and in patients on HD from morning peripheral venous blood samples, before the beginning of HD^13^. However, the influence of HD on the assessment of plasma metanephrines has not been studied to date. Importantly, it is not yet clarified if modifications of blood sampling would improve the diagnostic performance of measurements of plasma metanephrines in patients receiving HD. As there is a known arterio-venous gradient for plasma metanephrines in humans, sampling from the easily accessible shunt rather than from a vein might provide benefits as shunt blood closely matches arterial blood^14,15^. In addition, the time point of sampling may have a critical influence. During HD patients undergo a long period of rest that might be beneficial in terms of minimizing sympatho-adrenal activation^16^. On the other hand, the continued ultrafiltration has the potential to increase sympathetic activation during treatment^17,18^.

The aim of the study was, therefore, to establish optimal procedures for blood sampling to determine plasma concentrations of free metanephrines and 3-methoxytyramine in patients receiving HD.

## Methods

### Subjects

This study involved analysis of data from 107 patients receiving HD. Patients were enrolled under a multicenter prospective study at six clinical care centers^13^. The study was approved by local Ethics-Committees (Ethics Committee of the Technical University Dresden, Germany and Ethics Committee of the University Warsaw, Poland) and all patients provided written informed consents. All methods were performed in accordance with the relevant guidelines and regulations. All patients had an arterio-venous vascular access and were receiving HD or online hemodiafiltration for at least one year. Subjects were excluded if they presented with unstable conditions (sepsis or decompensated heart failure) or medication interfering with primary outcome parameters (tricyclic antidepressants, L-DOPA or medication containing sympathomimetic decongestants)^19^. Baseline characteristics of the participants are reported in Table 1.

**Table 1:** Characteristics of patients receiving hemodialysis (HD).

Number (n)	107
Female (%)	31.5
Age (years)	65.1 ± 14.3
BMI (kg/m^2^)	28.4 ± 7.5^a^
**Hypertension**	79.4%
Systolic blood pressure (mmHg)	137.7 ± 21.3^a^
Diastolic blood pressure (mmHg)	73.7 ± 12.3^a^
**Hypertensive patients using antihypertensives (%)**	98.1
Patients on beta-blocker (%)	83.5
Patients on ARBs/ACEi (%)	63.5
Diabetes type II (%)	38.3
ICT^c^ treatment (%)	26.0
**Causes of HD**	
Diabetic and hypertensive kidney disease (%)	51.4%
Chronic glomerulonephritis (%)	21.5%
Polycystic kidney disease (%)	10.3%
Systemic disease^d^ (%)	5.6%
Chronic pyelonephritis (%)	5.6%
Other^e^ (%)	5.6%
Hematocrit %	0.36 (0.26–0.44)^b^
GFR (mL/min)	7.3 ± 7.1^a^
Creatinine (µmoL/L)	745.9 ± 257.4
Urea (mmoL/L)	22 ± 5.4
Dialysis flow (mL/min)	300 (105–430)^b^
Plasma flow (mL/min)	192 (62–275)^b^
Clearance of metanephrines (mL/min)	78 (22–105)^b^

^a^Mean ± standard deviation.

^b^Median-range.

^c^*ICT* intensified insulin treatment.

^d^Systemic disease: HUS, amyloidosis, multiple myelom, lupus, vasculitis.

^e^Other: toxicity, medication, kidney tumor, hepatocardiorenal syndrome.

### Study design

The main goal of the study was to establish optimal conditions for blood sampling for measurements of plasma free metanephrines and 3-methoxytyramine in patients on HD. For this, two blood samples, one from the shunt and the other from a contralateral antebrachial vein, were collected from all patients after 30 min of supine rest before the start of HD and during the last hour of treatment. In a subgroup of 30 patients, ninety minutes after start of HD, the ultrafiltration rate was set to zero for 5 min and pre- as well as post-filter blood samples were drawn from the extracorporeal circuit to determine compound extraction. Patients remained in the semi-recumbent position throughout the HD. Duration of HD was approximately 4 h.

### Laboratory analysis

Measurements of plasma free NMN, MN and MTY concentrations were performed using LC–MS/MS^20^. All patients were instructed to fast and refrain from alcohol, nicotine, decaffeinated and caffeinated beverages for 12 h before the first sampling. A low amine diet was allowed during dialysis procedures prior to the second dialysis blood sampling, in a semi-recumbent position, whereas catecholamine containing foods were avoided. Blood samples were kept on ice until plasma was separated and stored frozen at − 80 °C before analyses.

### Statistical analysis

Statistical analyses utilized the JMP statistics software package (SAS Institute Inc, Cary, NC), with comparisons by Wilcoxon´s paired and Mann–Whitney U unpaired tests. Reference intervals for metabolites were established from distributions of the measured variables in populations with CKD receiving HD using non-parametric or parametric approaches as indicated by the nature of distributions. Upper cut-offs of reference intervals were determined by 97.5% percentiles of the distributions of each metabolite. Using the medians of the dialysis flow and the hematocrit, the plasma flow was calculated {= Blood Flow × (1 − Ht)}. From the dialysis plasma flow and the difference in concentrations of metanephrines before *vs* after the blood leaves the filter, the dialysis clearance was then calculated {= Plasma flow × [Cmetabolite_***beforefilter***_ − Cmetabolite_***afterfilter***_/Cmetabolite_***beforefilter***_]}.

## Results

### Patient characteristics

Hypertension was recorded in 79.4% of patients receiving HD and up to 98.1% received hypertension treatment. Up to 63.5% were treated with ACE inhibitors or ARBs and up to 83.5% with ß-blockers. Diabetes was present in 38.3% of patients. The proportion of patients treated with intensified insulin treatment (ICT) was 26%. Impaired renal function was mainly due to diabetic and hypertensive kidney disease, followed by glomerulonephritis and polycystic kidney disease (Table 1).

### Optimal conditions for blood sampling in patients on HD

#### Pre- versus end of dialysis

**Figure 1:**
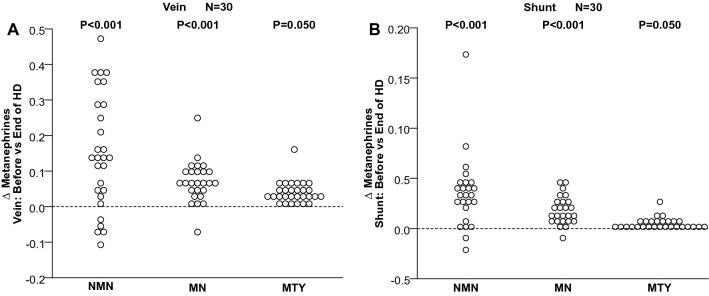
*Δ* of plasma concentrations of free normetanephrine (NMN), metanephrine (MN) and 3-methoxytyramine (3-MTY) before versus near the end of HD, in shunt (**A**) and in vein (**B**).

Normetanephrine and metanephrine concentrations, both in venous and shunt blood (Fig. 1A,B), were lower near the end than before dialysis (*P* < 0.0001). Similarly, concentrations of 3-methoxytyramine tended to be lower near the end than before dialysis, both in venous (*P* = 0.050) and shunt blood (*P* = 0.062), but failed to reach statistical significance (Fig. 1A,B).

#### Clearance of metanephrines and 3-methoxytyramine by the dialysis filter

**Figure 2:**
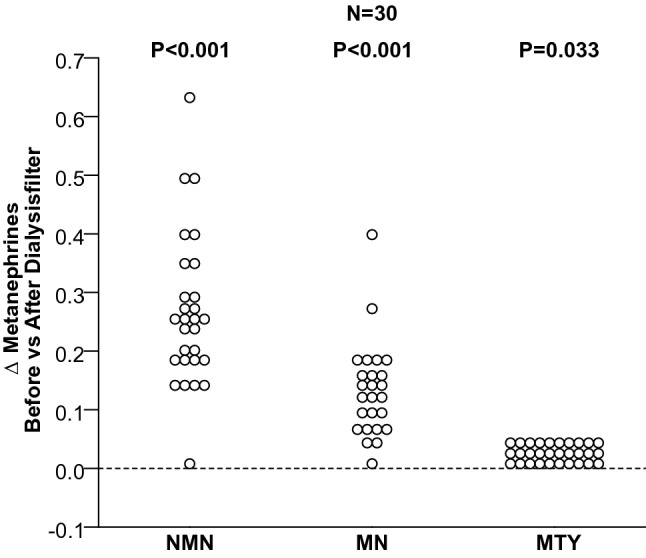
*Δ* of plasma concentrations of free normetanephrine (NMN), metanephrine (MN) and 3-methoxytyramine (3-MTY) before the blood enters the filter versus after leaving it.

Among patients receiving HD there were lower plasma concentrations of normetanephrine (*P* < 0.0001), metanephrine (*P* < 0.0001) and 3-methoxytyramine (*P* = 0.0014) in blood leaving than entering the dialysis filter (Fig. 2). The medians of dialysis clearance for NMN (79.7 mL/min) and MN (76.5 mL/min) were similar. The dialysis clearance of both metanephrines was, therefore, calculated to approximately 78 mL/min (Table 1), representing only 6.5% of the endogenous clearance of plasma metanephrines in patients with ESRD, which is assumed to reach 1200 mL/min^21,22^.

#### Shunt versus venous blood sampling

**Figure 3:**
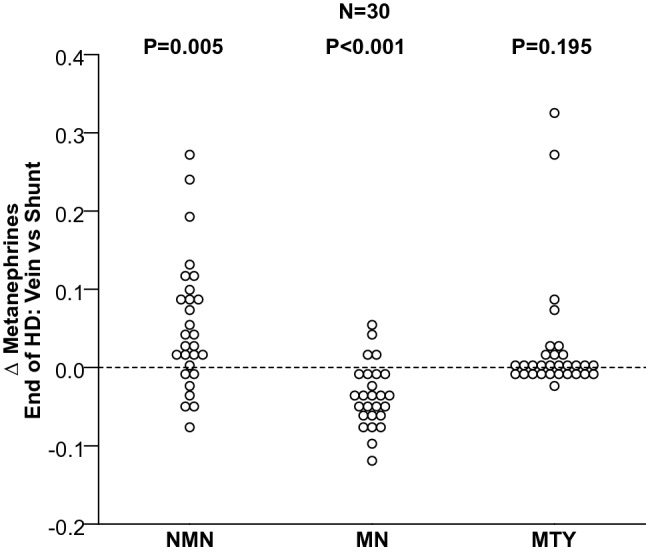
*Δ* of plasma concentrations of free normetanephrine (NMN), metanephrine (MN) and 3-methoxytyramine (3-MTY) in the venous versus shunt blood, near the end of HD.

For blood samples drawn near the end of dialysis, plasma normetanephrine concentrations among patients on HD were significantly lower (*P* = 0.005) and metanephrine concentrations higher (*P* < 0.0001) in shunt than in venous blood, with no significant difference for 3-methoxytyramine (Fig. 3).

### HD-specific reference intervals under optimal sampling conditions

Due to the high number of outliers involving remarkably high plasma concentrations of 3-MTY in patients with ESRD, HD-specific cut-off values are recommended only for plasma metanephrines. Compared to already established stage IV/HD specific reference intervals from the vein before HD^13^, upper cut-off levels (97.5% percentiles of reference intervals) for NMN in patients on HD were 34% lower when the blood was drawn from the shunt near the end of HD than from the vein before HD. In contrast, upper cut-off levels for MN were 12% higher when the blood was drawn from the shunt near the end of HD than from the vein before HD (Table 2).

**Table 2:** HD specific cut-offs (97.5% percentiles) for plasma metanephrines for patients on HD when the blood is drawn from the vein and before HD versus the shunt, near the end of HD.

	Patients on HD	
	Vein before HD	Shunt near the end of HD
**Normetanephrine (nmol/L)**		
Median	0.649	0.451
2.5 Percentile	0.181	0.131
97.5 Percentile	1.622	1.055
**Metanephrine (nmol/L)**		
Median	0.231	0.191
2.5 Percentile	0.092	0.064
97.5 Percentile	0.417	0.472
**Methoxytyramine (nmol/L)**		
Median	0.075	0.043
2.5 Percentile	0.019	0.007
97.5 Percentile	0.896	0.653

## Discussion

The present study establishes that measurements of plasma metanephrines in patients receiving HD are most appropriate using blood drawn from shunt at a time near the end of dialysis (Box 1). Moreover, this study outlines specific upper cut-offs of reference intervals for plasma metanephrines under these most optimal conditions in patients on HD that can be expected to minimize false positive results.

The diagnostic work-up of patients suspected of harboring a PPGL among patients on HD is a clinical challenge. Consequently, reliable biochemical tests to exclude or confirm these tumors are crucial. Urinary tests are clearly unreliable for patients on HD^5,6^, while for plasma free metanephrines the reported prevalence of false positive results had varied from 85% using high pressure liquid chromatography-electrochemical detection (HPLC-ED)^12^ to approximately 10.2% for NMN and 14% for MN when using LC–MS/MS, confirming the superiority of this method, particularly in terms of relative freedom of analytical interferences^13^. However, the remaining relatively high prevalence of false positive results for either elevation of NMN or MN likely reflects chronic activation of the sympathetic nervous system, which characterizes patients with ESRD^8,9^. The kidneys are responsible for 14 to 16% of the circulatory clearance of plasma free metanephrines^21^, so that some smaller increase in plasma concentrations can also be expected to result from impaired renal function.

The above considerations highlight the need for optimized pre-analytical procedures and specific upper limits of reference intervals under most optimal sampling conditions. With recognition of the above needs, the main aim of our study was to establish the most appropriate sampling conditions for measurements of plasma metanephrines and 3-MTY during HD. Due to the high number of outliers involving remarkably high levels of 3-MTY in patients on HD, optimized pre-analytical procedures and specific upper cut-offs were focused on plasma metanephrines. The lower plasma concentrations of metanephrines, both in venous and shunt blood, near the end rather than before starting HD indicate the former time point as preferable to minimize false positive results. The prolonged recumbency (4 h HD duration) could provide one explanation^16^ while an effect of the dialysis filter to increase clearance of metanephrines likely also contributes to the lowered concentrations towards the end of dialysis. Nevertheless, fractional extractions of metanephrines by the dialysis filter were calculated to contribute to less than 7% of the endogenous clearance of metanephrines in patients on HD.

In addition to showing that the last hour of dialysis is the most appropriate time for blood sampling, the present study also established the shunt as the best sampling site. This conclusion was based on the findings that plasma concentrations of NMN were lower and those of MN higher in shunt than in venous blood. The latter observation can be explained by extraction of this metabolite during passage from arterial to venous sites resulting in a physiologic arterio-venous concentration gradient^21,23,24^. NMN is also extracted during passage from arterial to venous sampling sites, but, in contrast to MN, it is also generated by metabolism of locally produced norepinephrine^10^. As false positive results are much more prevalent for NMN and the same metabolite is the most important for the diagnosis of PPGLs, the dialysis shunt represents the most appropriate sampling site to both minimize false-positive results for NMN, and detect any signal for both metabolites from catecholamine-producing tumors.

Our study, like all previous studies, has limitations. The present study did not include patients on HD with PPGLs, as such patients are extremely rare. Another limitation is that we can not definitely exclude the possibility that any of our patients actually harbors a PPGL. If some of our patients, though, would harbor a PPGL, one would expect that this would be uncovered by the development of signs and symptoms related to tumoral catecholamines. All the patients had clinical follow up for at least 4 years, and none of them showed any signs of PPGLs. We, therefore, assume that the presence of a tumor in some of the patients of our study is extremely unlikely.

A final limitation of our study is that we did not include patients on continuous ambulatory peritoneal dialysis (CAPD) or on “pure” hemofiltration. Patients on CAPD do not usually have arterio-venous fistula and “pure” hemofiltration is not a common type of dialysis among patients with end stage renal disease. In our cohort, though, apart from patients with HD we have included twelve patients on online-hemodiafiltration, who showed no significant differences in pre-, post-filter or near the end of HD concentrations of plasma metanephrines, compared to patients on HD (Supplementary figures). In that respect, we assume that convective solute and diffuse transport modify metanephrine levels in a similar way.

## Conclusion

This study is important in being the first to comprehensively address optimal sampling conditions using blood from the dialysis shunt near the end of HD with optimal reference intervals for plasma metanephrines for the diagnosis of PPGLs among patients on HD. Our study thereby provides immediate guidance to clinicians in daily routine practice.

## Supplementary Information

Supplementary Information 1.

Supplementary Information 2.
