# Supplementary material for: Optimized procedures for testing plasma metanephrines in patients on hemodialysis
Source: Sci Rep. 2021 Jul 19;11:14706. doi: 10.1038/s41598-021-94104-9 (PMC8290036; doi:10.1038/s41598-021-94104-9)
Supplement: Supplementary file 1 — Supplementary Information 1. [file 41598_2021_94104_MOESM1_ESM.pptx]

## Slide 1
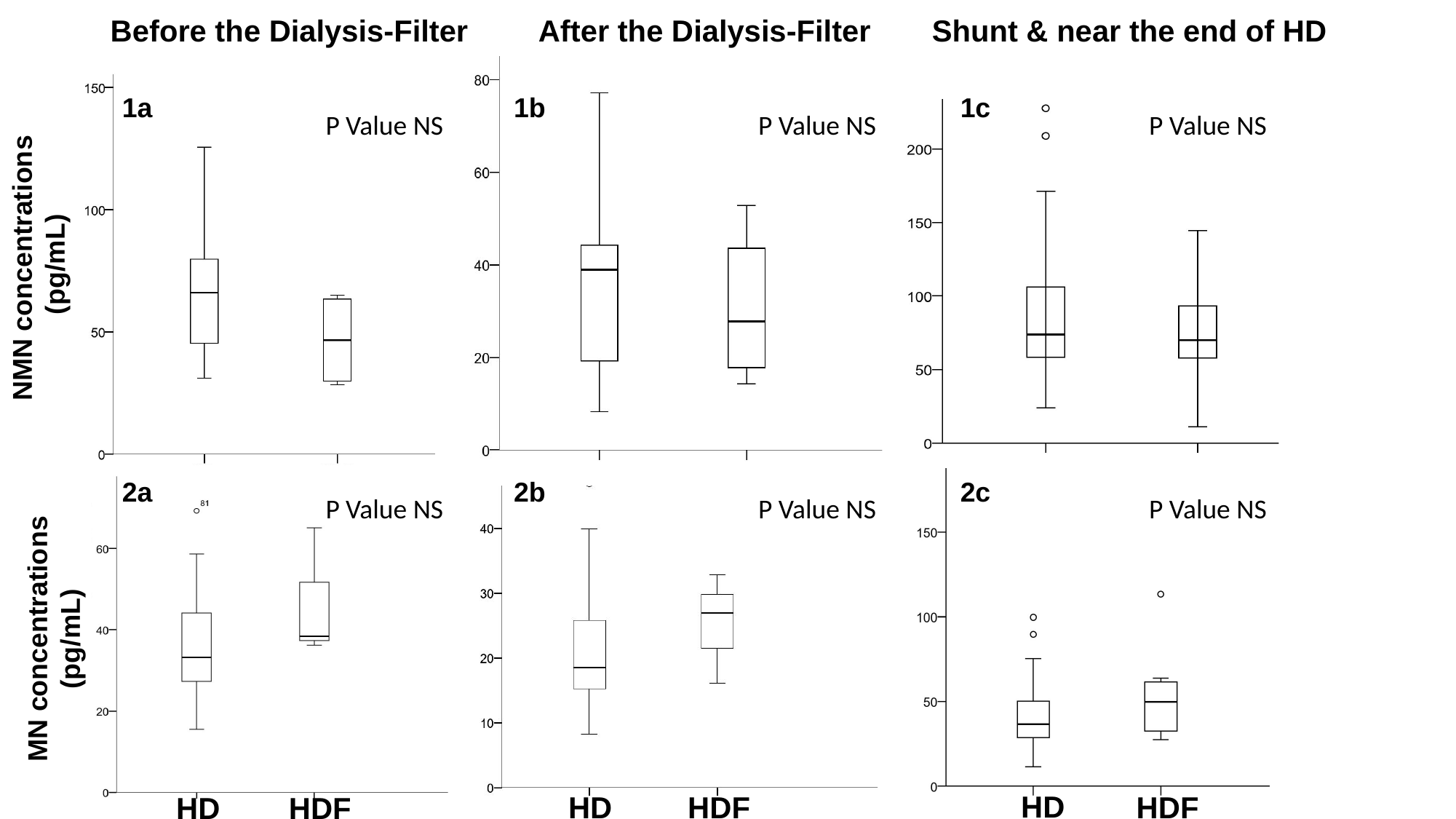

Before the Dialysis-Filter
After the Dialysis-Filter
Shunt & near the end of HD
1a
1b
1c
P Value NS
P Value NS
P Value NS
NMN concentrations (pg/mL)
2a
2b
2c
P Value NS
P Value NS
P Value NS
MN concentrations (pg/mL)
HD
HD
HDF
HDF
HD
HDF

## Slide 2
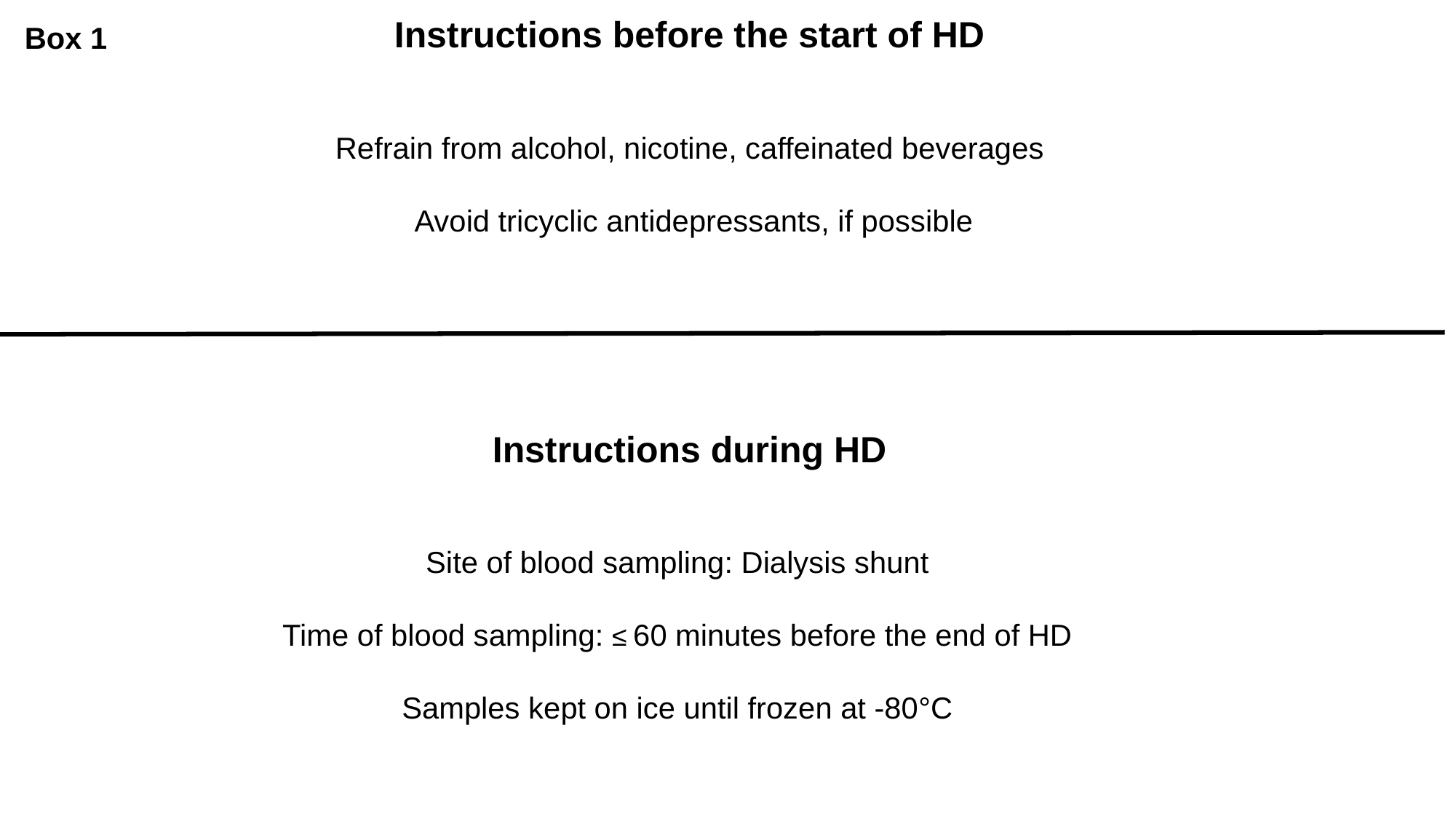

Instructions before the start of HD
Box 1
Refrain from alcohol, nicotine, caffeinated beverages
 Avoid tricyclic antidepressants, if possible
Instructions during HD
Site of blood sampling: Dialysis shunt
Time of blood sampling: ≤ 60 minutes before the end of HD
Samples kept on ice until frozen at -80°C
