# Supplementary material for: Optimized procedures for testing plasma metanephrines in patients on hemodialysis
Source: Sci Rep. 2021 Jul 19;11:14706. doi: 10.1038/s41598-021-94104-9 (PMC8290036; doi:10.1038/s41598-021-94104-9)
Supplement: Supplementary file 2 — Supplementary Information 2. [file 41598_2021_94104_MOESM2_ESM.docx]

**Supplementary legends**

**Box 1**. Instructions for optimal conditions for testing plasma metanephrines in patients on HD.

**Supplementary Figures**. Comparison of normetanephrine (NMN) (Fig 1) and metanephrine (MN) (Fig 2) concentrations between patients on hemodialysis (HD) and on online-hemodiafiltration (HDF), before the dialysisfilter (a), after the dialysisfilter (b) and at the shunt, near the end of HD (c).
